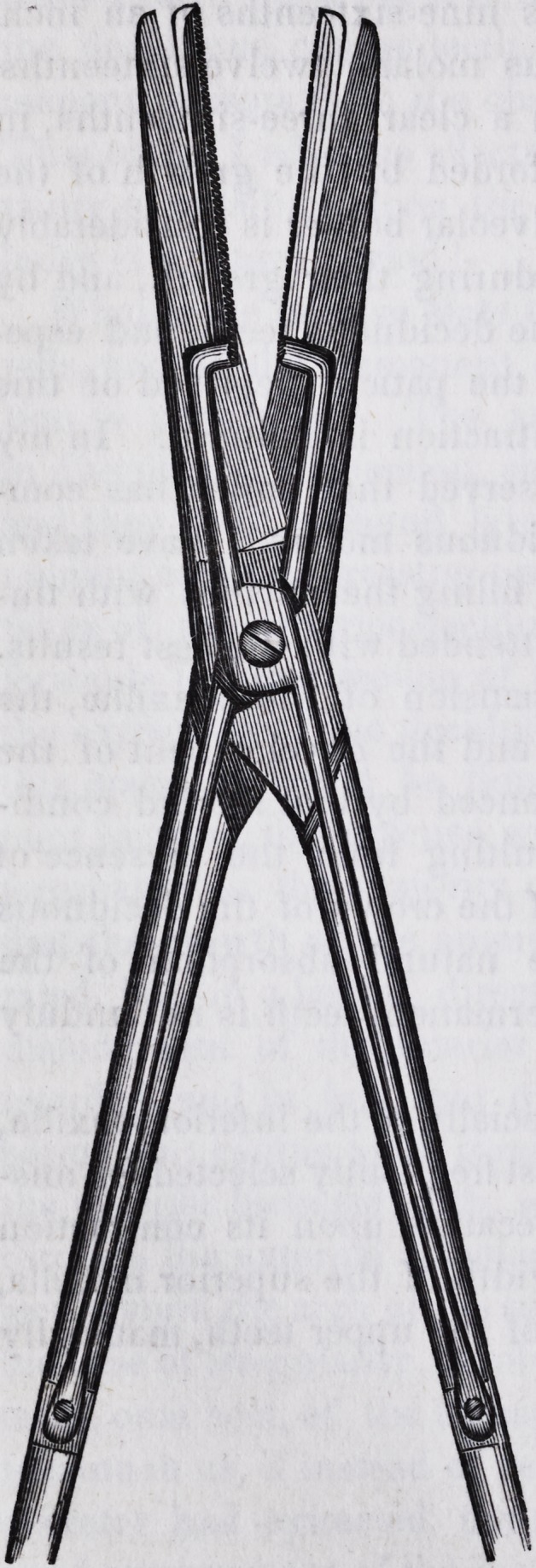# Uvula Scissors, a New Instrument

**Published:** 1847-03

**Authors:** S. P. Hullihen

**Affiliations:** Wheeling, Va.


### ARTICLE VIII.

*Uvula Scissors, a New Instrument.* By DR. S. P. HULLIHEN, of Wheeling, Va.

THE accompanying cut represents the construction of this instrument pretty well, except a slight lateral curvature that is in the blades. It likewise exhibits so plainly the adaptation of the instrument to the purpose for which it is designed, that a further description of it, or any directions as to the manner of using it, is deemed entirely unnecessary.

This instrument was contrived some time during the winter of 1843, at which time, through the aid of Mr. Kryter, of this city, I had one constructed; after testing it fully, and finding that it fulfilled every indication necessary for the easy and speedy removal of just so much of the uvula as might be desired, I placed it in the hands of Wm. R. Goulding, surgical instrument maker, of New York, some time during the month of July, 1845. This gentleman immediately made up several of them, and has kept the instrument for sale ever since.

Although both useful and convenient, this instrument is not claimed to be one of much importance, simply because the operation for which it is intended is but a very trifling one. Yet, unimportant as the instrument may be, I perceive that it has been exhibited before the Dublin Surgical

Society, with some show of consequence, and claimed as the invention of a Mr. Carte. It was presented before that Society by Dr. Beatty, who, after having exhibited a new instrument for dividing the frænum linguæ, said, "The next instrument he had to exhibit, displayed, he observed, much greater ingenuity, and is the invention of Mr. Carte, who, in order to have justice done to the instrument, ought himself to have submitted it for the inspection of the Society. It had been placed in his (Dr. Beatty's) hands by Mr. Millikin, by whom it had been constructed.

"The instrument he looked on as an exceedingly ingenious contrivance for facilitating the removal of a portion of the uvula, an operation which, though simple, is yet sometimes attended with difficulty, and occasionally with danger too, on account of the detached portion falling into or against the air passages. This instrument, at the same time that it cuts the uvula, has a provision by which it secures the divided part, and thereby prevents the possibility of the inconvenience just alluded to; it consists, essentially, of a pair of scissors with blunt points, but, in addition to the ordinary cutting blades, there are, beneath these, attached by means of screws, a pair of blunt supplementary blades, whose flat surfaces come in contact with the substance of the uvula, and seize it while it is being detached by the cutting blades."—*Dublin Med. Press, May* 13, 1846, p. 290.*

Now, as this instrument has gone forth to the profession in Europe, as the invention of Mr. Carte, and in this country as my invention, and as it must doubtless appear highly improbable to every one who may have noticed this, that an instrument alike in every particular, should have been original with us both, and being unwilling to appear as having owned that which did not properly belong to me, I thought it right to place the claims of both Mr. Carte and myself, so far as it was in my power, before the profession, as the best way of explaining this singular coincidence.

This instrument, when made a few sizes larger than represented by the cut, and the supplementary blades with long sharp teeth, likewise answer a most excellent purpose for the excision of enlarged tonsils.

* Braithwaite's Retrospect, part thirteenth, p. 245.

## Figures and Tables

**Figure f1:**